# Liposome-Encapsulated Botulinum Toxin A in Treatment of Functional Bladder Disorders

**DOI:** 10.3390/toxins14120838

**Published:** 2022-12-01

**Authors:** Fan-Ching Hung, Hann-Chorng Kuo

**Affiliations:** 1 Department of Urology, National Taiwan University Hospital Yunlin Branch, Douliu 64041, Taiwan; 2 Department of Urology, Hualien Tzu Chi Hospital, Buddhist Tzu Chi Medical Foundation, Tzu Chi University, Hualien 97004, Taiwan

**Keywords:** botulinum toxin A, liposome, bladder oversensitivity, interstitial cystitis, detrusor overactivity

## Abstract

Botulinum toxin A (BoNT-A) intravesical injections have been used to treat patients with refractory functional bladder disorders such as overactive bladder (OAB) and interstitial cystitis/bladder pain syndrome (IC/BPS), but the risk of adverse events and the need for repeated injections continue to prevent widespread application of this treatment. Liposomes are vesicles that comprise concentric phospholipid layers and an aqueous core; their flexible compositions enable them to adsorb and fuse with cell membranes and to deliver drugs or proteins into cells. Therefore, liposomes have been considered as promising vehicles for the less invasive delivery of BoNT-A. In previous placebo-controlled trials including patients with OAB refractory to medical treatment, it was shown that liposomal BoNT-A could significantly decrease the frequency and urgency of urination. In patients with IC/BPS, it was shown that liposomal BoNT-A could also improve bladder pain, but the therapeutic efficacy was not superior to that of the placebo. As the therapeutic mechanisms of BoNT-A include the decreased expression of nerve growth factors, P2X3 receptors, and vanilloid receptors on C-fibers, liposomal BoNT-A might play a more promising role in the treatment of bladder oversensitivity. This article features the contemporary literature regarding BoNT-A, liposomes, and liposomal BoNT-A treatment for functional bladder disorders and potential clinical applications in the future.

## 1. Introduction

Functional bladder disorders are a group of lower urinary tract disorders with unclarified structural etiologies, including overactive bladder (OAB) syndrome, bladder hypersensitivity, and interstitial cystitis/bladder pain syndrome (IC/BPS). These disorders are characterized by a relapsing–remitting course and require multiple treatments [1].

The prevalence rate of OAB ranges from 10.8% to 27.2% in men and 12.8% to 43.1% in women [2,3,4,5,6]. OAB is characterized by frequency, urgency, and nocturia, with or without urgency urinary incontinence (UUI). Behavioral therapies, oral antimuscarinics, and oral β3-adrenoceptor agonists are often offered as the first- and second-line treatments [7]. However, there are high withdrawal rates when using these OAB medications due to unsatisfactory symptom control or undesirable adverse effects [8].

Population-based studies conducted in the USA revealed that the prevalence of IC/BPS is 2% to 4.2% in men and 2.7% to 6.3% in women [9,10]. The definition of IC/BPS according to the Society for Urodynamics and Female Urology (SUFU) is the perception of pain, pressure, or discomfort in the urinary bladder caused by lower urinary tract symptoms lasting for over six weeks, without other identifiable causes [11]. American Urological Association (AUA) guidelines have suggested behavior modification; pain management; specialized manual physical therapy; oral agents such as amitriptyline, cimetidine, hydroxyzine, and pentosan polysulfate sodium; and intravesical therapy including dimethylsulfoxide (DMSO), heparin, and lidocaine as initial therapeutic approaches [12].

Intravesical botulinum toxin subtype A (BoNT-A) injections have been considered as an effective treatment option for patients with OAB and IC/BPS refractory to conventional medications or therapies. However, the risk of adverse events after BoNT-A injection and the need for repeated injections continue to prevent the widespread application of this treatment [13,14]. Clinicians have been searching for a less invasive treatment modality for the delivery of BoNT-A to achieve treatment efficacy without intravesical injection and adverse events [15].

Liposomes are vesicles that comprise concentric phospholipid layers and an aqueous core; their flexible compositions enable them to adsorb and fuse with cell membranes and deliver drugs or proteins into cells. Therefore, liposomes have been considered as promising vehicles for the less invasive delivery of BoNT-A to achieve a therapeutic effect without intravesical injection or the development of adverse events. In this article, we aim to review the current literature regarding the management of functional bladder disorders via liposomal BoNT-A instillation.

## 2. Botulinum Toxin A Mechanism and Clinical Applications

### 2.1. The Urinary Bladder and Botulinum Toxin A Mechanism

The bladder wall comprises urothelium, detrusor muscle, and adventitia, from the lumen to the outer surface. The bladder urothelium serves as an impermeable barrier preventing the penetration of urine and waste content into the submucosal layer [16]. From the apical to the detrusor side, the urothelium is constituted of umbrella cells, intermediate cells and basal cells [17]. Tight junctional proteins, uroplakins, and a glycosaminoglycan (GAG) mucin layer cover the umbrella cells, which help to establish the barrier function.

Botulinum toxin is a neurotoxic protein produced from Clostridium botulinum; the subtype A (BoNT-A) is the most popular clinically used form, with a 50 kDa light chain and a 100 kDa heavy chain bridged by a disulfide bond [18]. The heavy chain of BoNT-A binds to synaptic vesicle glycoprotein 2 (SV2) receptors on the surface of the parasympathetic nerve terminal and is then endocytosed into synaptic vesicles. The light chain is released from the vesicle to cleave synaptosomal-associated protein, 25 kDa (SNAP-25) and prevents the vesicles from fusion with the nerve terminal membrane, thereby inhibiting acetylcholine release and detrusor muscle contraction [19]. The accumulation of BoNT-A can decrease the bladder sensation by inhibiting ATP release into the suburothelium, indicating its mechanism of action may also involve inhibition of neurotransmitter release from afferent nerve terminals and the urothelium [20].

### 2.2. Botulinum Toxin A Injection for OAB

BoNT-A has been commonly utilized to treat bladder muscular hypercontractility and modulate sensory and inflammatory function [21]. In the last decade, the FDA approved the intradetrusor injection of 200 units of BoNT-A for the treatment of neurogenic detrusor overactivity (NDO) and the injection of 100 units of BoNT-A for the treatment of idiopathic OAB [22]. It was shown that BoNT-A 100 U intradetrusor injections significantly improved all OAB symptoms, including urgency, UUI, and health-related quality of life [23,24,25]. It was found that male gender, a baseline post-void residual (PVR) volume of more than 100 mL, and medical comorbidity are independent risk factors of acute urinary retention or large PVR after intravesical BoNT-A injections for the treatment of idiopathic OAB [26]. Subjectively successful treatment outcomes of intravesical BoNT-A injection for patients with OAB were associated with improvements in OAB symptoms but not with increases in bladder capacity, PVR volume, or voiding efficiency [27]. The balance of the therapeutic and adverse effects of BoNT-A injections can be modified by amending the dose and changing the injection site [28]. It was shown that BoNT-A injection at the bladder base and trigone could relieve the sensation of urgency but did not increase the PVR volume or the bladder capacity. After intravesical BoNT-A injections, the duration of their effect on OAB symptoms is about 6–9 months; therefore, repeated injections are necessary to maintain the efficacy of this treatment [29].

### 2.3. Botulinum Toxin A Injection for IC/BPS

The clinical symptoms of IC/BPS, such as frequency, urgency, and bladder pain, are considered to result from urothelial dysfunction and increased urothelial permeability [30]. There are higher levels of urothelial cell apoptosis, mast cell activation, abnormal E-cadherin expression, and less cell proliferation in patients with IC/BPS [31]. Increased urinary nerve growth factor levels were noted in patients with IC/BPS, and decreased levels in successful BoNT-A treatment responders, suggesting neurogenic inflammation might be involved in the pathogenesis of IC/BPS [32]. Intravesical injection of BoNT-A followed by cystoscopic hydrodistention significantly improved the clinically successful treatment response rate compared with cystoscopic hydrodistention alone [33]. Repeated intravesical BoNT-A injections in patients with IC/BPS resulted in significantly reduced numbers of apoptotic cells and the activation of mast cells [34]. The immunohistochemical findings were associated with the improvement of the maximal bladder capacity and glomerulation grade after cystoscopic hydrodistention. Patients who received repeated BoNT-A injections experienced an increase in functional bladder capacity and had longer-term pain relief than the relief that a single injection provided, without the increased prevalence of adverse events [35]. In patients with refractory IC/BPS, compared with the placebo-controlled group, suburothelial injections of 100 U of BoNT-A plus cystoscopic hydrodistention significantly reduced bladder pain symptoms [14]. In the AUA guidelines for IC/BPS, it is recommended to administer intradetrusor BoNT-A injections if other treatments provide an inadequate improvement of symptoms, while patients should be informed of the possibility of intermittent self-catheterization. In real-life practice, there is very little need for intermittent self-catheterization after BoNT-A injection for patients with IC/BPS [14,33,35]. Evidence showed that a reduced morbidity rate was reported with the dose of 100 U [12].

## 3. Liposome Mechanism and Applications to Functional Bladder Disorders

Urothelium serves as a barrier that prevents urine constituents and solutes from penetrating into the submucosal layer. Substances move across the urothelium via one pathway through cells and another through tight junctions and lateral intercellular spaces [17]. Alterations in either cellular or tight junction permeability change the urothelium barrier’s characteristics [36]. Liposomes are microlevel vesicles that consist of concentric phospholipid layers and an aqueous core, with the ability to adsorb and merge with cells. Their flexible compositions make them suitable delivery vehicles for various molecules, including proteins, nucleotides, and small drug molecules [16]. Liposomes not prepared with drugs could form a molecular film on cell surfaces, and their wound-healing properties on skin were confirmed with animal models [37,38].

In a rat model of protamine-sulfate/potassium-chloride-induced bladder hyperactivity, it was shown that the intravesical instillation of liposomes could reverse the high micturition frequency [39]. Intravesical liposome infusion significantly reversed the decrease in the intercontractile interval in rats with chemically induced bladder hyperactivity, showing superior beneficial effects compared to DMSO and pentosan polysulfate sodium [40]. It was hypothesized that liposomes might reinforce the barrier function of a leaky urothelium and gain resistance against the penetration of irritants. In a comparative study, it was shown that the intravesical instillation of liposomes could achieve similar efficacy to oral pentosan polysulfate sodium for patients with IC/BPS. The instillation of 80 mg of liposomes in 40 mL of distilled water once weekly for 4 weeks was shown to improve the symptoms of pain and urgency for up to 8 weeks [41]. In a study comparing once-a-week or twice-a-week treatment within a 4-week period, 6 of 12 patients and 4 of 5 patients responded to liposome treatment, respectively. More frequent instillation was tolerable and had a potential benefit with regard to symptom flare-up, while the effects after 8 weeks of follow-up were not clear [42]. An open-label clinical evaluation also revealed symptom improvement without treatment-related adverse events [43].

## 4. Mechanism and Clinical Effects of Liposome Encapsulated Botulinum Toxin A

Although the therapeutic effects of intravesical BoNT-A injections on OAB and IC/PBS have been established, the need for a novel delivery method with lower risk became apparent due to certain adverse effects related to these injections, such as hematuria, injection pain, urinary tract infection, the uneven distribution of the drug, and drug leakage outside the bladder [44,45]. Thus, intravesical instillation was proposed with potential advantages, such as extending duration of drug contact with urothelium, decreasing systemic toxicity side effects while achieving higher drug concentrations, and modulating urothelium repair, neurotransmission, and sensory nerve function [46].

**Figure 1:**
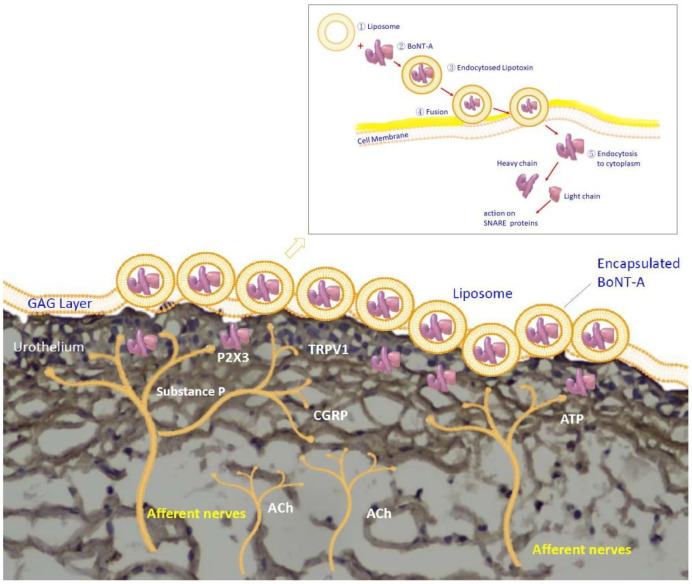
Empty liposomes mixed with botulinum toxin A (BoNT-A) solution yields encapsulated BoNT-A. After fusion with the phospholipid layer of the cell membrane of urothelial cells, the BoNT-A protein is endocytosed and transferred into the cytoplasm of the urothelial cells. The BoNT-A protein is cleaved into a heavy chain and a light chain; the latter acts on the SNARE protein complex, inhibits the releases of neurotransmitters, including acetylcholine (ACh), adenosine triphosphate (ATP), calcitonin-gene-related peptide (CGRP), and substance P, from the sensory nerve terminals, and suppresses the expression of transient receptor potential vanilloid receptor subfamily 1 (TRPV1) as well as purinergic receptor P2X3.

The instillation of BoNT-A delivery via liposomes was first reported in a rat model [47]. Rats pretreated with liposomes and BoNT-A displayed a considerable decrease in the intercontractile interval after acetic acid (AA) infusion, whereas those pretreated with liposome-encapsulated BoNT-A (liposomal BoNT-A) showed a significantly diminished response to AA instillation. The study results demonstrated that liposomal BoNT-A pretreatment could suppress AA-induced bladder overactivity. Histologically, less inflammatory cell accumulation and edematous changes were also observed in the liposomal BoNT-A pretreated group. The calcitonin-gene-related peptide (CGRP) is one of the sensory mediators that is released in response to toxic stimuli, and BoNT-A can inhibit its release [48]. CGRP immunostaining in the bladder mucosal layer revealed that intravesical liposomal BoNT-A instillation inhibited CGRP release from afferent nerve terminals. The expression of SNAP-25 was also significantly decreased in the liposomal BoNT-A pretreated group compared to that in the liposome or BoNT-A pretreated groups, indicating that liposomal BoNT-A pretreatment could cleave SNAP-25. The concept of using liposomes as vehicles for BoNT-A delivery was supported by these results, while the actual mechanism of liposomal BoNT-A adsorption and transport in the urothelium remains to be discovered (Figure 1).

### 4.1. Liposome-Encapsulated Botulinum Toxin A for Treatment of OAB

The liposome encapsulated BoNT-A for clinical use was prepared with the following procedures. Sphingomyelin mixed with normal saline (N/S) creates a liposomal dispersion of sphingomyelin. Sphingomyelin liposomes are available for preparation at a concentration of 2 mg/mL (2.84 mM) in N/S containing 500 mM KCl (LP-08, Lipella Pharmaceuticals Inc., Pittsburgh, PA, USA). Lipotoxin was prepared before application by hydrating 80 mg freeze-dried LP-08 in 40 mL N/S and 200 U BoNT-A (Botox, Allergan, Irvine, CA, USA) in 10 mL N/S to make a total volume of 50 mL at room temperature [49].

In a double-blind, randomized, controlled trial, 24 patients with OAB received the intravesical instillation of liposomal BoNT-A (80 mg of liposomes and 200 U BoNT-A) or N/S (control group) [49]. The liposomal BoNT-A treatment was effective in reducing the number of frequency and urgency episodes 1 month after treatment, with an efficacy of 50% (6 out of 12 patients). No adverse events, such as urinary retention, large PVR volumes, or urinary tract infections (UTIs), were reported during the study period. In immunohistochemical staining and Western blotting, there were no significant differences in the synaptic vesicle protein 2A (SV2A) and SNAP-25 expressions at baseline and 3 months after liposomal BoNT-A treatment in the responders or non-responders. Another multicenter, double-blind, randomized, placebo-controlled study enrolled 62 patients with OAB inadequately treated with antimuscarinics to receive an intravesical instillation of liposomal BoNT-A or normal saline [50]. The results showed that treatment with liposomal BoNT-A reduced the number of episodes of voiding frequency, urgency, and overactive bladder symptom score (OABSS) but not urge incontinence 1 month after treatment. No urinary retention, increase in PVR, or any drug-related adverse events were noted. A study comparing the BoNT-A injection and liposomal BoNT-A instillation showed that there was decreased expression of ionotropic purinergic receptor P2X3 in the urothelia of responders, but no cleaved SNAP-25 was detected in the suburothelium 1 month after liposomal BoNT-A treatment [51]. To further enhance its clinical usability, the short therapeutic duration and depth of penetration require technical improvement in terms of instillation.

### 4.2. Liposome-Encapsulated Botulinum Toxin A for IC/BPS

A multicenter, double-blind, randomized, placebo-controlled study enrolled patients with refractory IC/BPS. In total, 96 eligible patients were assigned at a 1:1:1 ratio to three groups, including treatment with liposomal BoNT-A (BoNT-A 200 U with 80 mg liposome), BoNT-A 200 U in normal saline, or normal saline alone. Four weeks after treatment, liposomal BoNT-A instillation was associated with significant decreases in O’Leary–Sant symptom scores (including Interstitial Cystitis Problem Index [ICPI] and Interstitial Cystitis Symptom Index [ICSI]), and the visual analog scale for pain, as well as an increase in the global response assessment. However, no differences in the improvement rates among the three groups were found, suggesting a significant placebo effect might have existed in this study [52]. IC/BPS has multifactorial etiologies, including urinary, infection, organ-specific, neurological/systemic, tenderness, and psychosocial domains [53]. Multimodal treatment strategies and modified liposomal BoNT-A instillation concentrations may be necessary to provide more convincing outcomes in a future study.

### 4.3. Liposome-Encapsulated Botulinum Toxin A for Bladder Oversensitivity

Several sensory receptors have been found to be expressed in the bladder urothelial and suburothelial nerves, including substance P, P2X3, the transient receptor potential vanilloid receptor 1 (TRPV1), and the CGRP [54,55,56]. These receptors participate in the transmission of bladder sensation during the urine storage phase [57]. These sensory receptors are co-localized on the sensory fibers and urothelium. Capsaicin or its ultrapotent analog, resiniferatoxin, treatment targeting TRPV1 desensitization had been found to effectively decrease bladder oversensitivity and OAB symptoms [58,59,60,61,62]. Recent studies also revealed that acetylcholine (ACh) and ATP are involved in bladder filling and fullness transmission in response to the stretch of the bladder [63]. The release of ACh and ATP from urothelial cells increases with aging, which might be associated with the increased incidence of bladder oversensitivity and OAB in older people [64].

BoNT-A has both sensory and motor effects in treating patients with detrusor overactivity and OAB. Decreases in the expression of TRPV1 and P2X3 on the suburothelial sensory afferents were found after detrusor BoNT-A injections [27,65]. It was found that patients also experience reductions in the frequency and urgency of urination after BoNT-A injection [23]. Reduced TRPV1 and P2X3 receptor expressions on the suburothelial afferent nerves are likely to result in bladder oversensitivity alleviation. Bladder inflammation is also frequently found in patients with OAB, IC/BPS, and oversensitivity with symptoms of urgency and frequency [66]. Chronic neural plasticity due to unresolved bladder inflammation and sensory receptor activation may increase afferent activity by influencing antinociceptive activity and cause increases in nerve growth factor (NGF) levels and bladder oversensitivity [67,68].

As the pathophysiology of bladder oversensitivity involves chronic inflammation, neural hyperactivity, and sensory receptor overexpression, the intravesical instillation of liposomal BoNT-A might effectively inhibit the progress of these sensory receptors’ expressions and subsequent neuroplasticity, further lowering the threshold of bladder sensation and hypersensitivity [69]. The weakness of liposomal BoNT-A in managing OAB and IC/BPS might become the strength of treatment for bladder oversensitivity, because the penetration of BoNT-A is limited to the urothelium without affecting detrusor contractility.

## 5. Future Perspectives for Liposome-Encapsulated Botulinum Toxin A for Functional Bladder Disorders

Liposomes can serve as vehicles to deliver BoNT-A across the cell membrane of the urothelium and may act on afferent nerves to reduce pain and inflammatory processing without directly impacting the detrusor. Based on previous studies, the intravesical instillation of liposomal BoNT-A could ameliorate lower urinary tract symptoms in patients with functional bladder disorders [70]. In addition to future studies, which are expected to reinforce the clinical efficacy in patients with OAB and IC/BPS, the potential expansion of liposomal BoNT-A can also be explored in further research.

Recent studies have explored the underlying pathophysiology of OAB and IC/BPS. Chronic inflammation and ischemic change in the urinary bladder might underlie these functional bladder disorders [71]. Due to the presence of chronic inflammation, urothelial cell proliferation, differentiation, and maturation are impaired, resulting in defective urothelia in IC/BPS bladders and the increased sensory expressions of the bladders of individuals with OAB [31,72]. As liposomal BoNT-A can only deliver the BoNT-A protein to the upper part of bladder urothelium, it is not likely this treatment will have an effect on the urotheliogenic OAB, but not on neurogenic, musculogenic, or central-nervous-system-related OAB. On the other hand, there are many different subtypes of IC/BPS that might result in different phenotypes with small or large maximal bladder capacities and different grades of glomerulations [73]. Treatment with liposomal BoNT-A can provide benefits to patients with IC/BPS who solely have urothelial dysfunction without bladder wall inflammation. For patients with IC/BPS who have small bladder capacities and Hunner’s lesions, which are characterized by confined, crimson mucosal area with small vessels radiating toward a central scar [74], liposomal BoNT-A might not be an effective treatment. With this in mind, by carrying out patient selection using clinical presentation as well as urinary biomarkers to identify urotheliogenic OAB and pure urotheliogenic IC/BPS, we might effectively choose suitable patients with OAB or IC/BPS for this novel treatment [75].

Furthermore, although liposomes can encapsulate BoNT-A proteins and deliver the proteins across the cell membrane, their efficacy might be limited by the instillation time, the bladder volume during instillation, and the dose of BoNT-A. Preliminary data have shown that 200 U BoNT-A plus liposomes can have limited clinical efficacy in patients with OAB or IC/BPS, possibly due to this treatment’s limited penetration depth into the urothelium [49,51]. If we increase the dose of BoNT-A and instillation duration to facilitate more liposomal BoNT-A to penetrate across the cell membrane, the therapeutic effect might be better than what has currently been shown. A recent study also revealed that applying low-energy shock waves on the bladder can increase the bladder urothelial permeability [76]. Pretreatment with low-energy shock waves might also enhance the penetration of liposomal BoNT-A into the urothelium and achieve a better treatment outcome. The classification of OAB and IC/BPS subtypes is important for the selection of suitable patients for the treatment [77].

BoNT-A has been utilized in the treatment of functional bladder disorders for over 20 years, although the limited number of licensed applications has prevented its clinical popularity. With various pharmacologic mechanisms, including inhibition of the release of neuropeptides, neuromodulation, and anti-inflammatory and anti-sense actions, BoNT-A can be an alternative treatment for lower urinary tract dysfunctions that are refractory to conventional medications or surgical procedures [78]. Detrusor BoNT-A injections could provide therapeutic effectiveness with regard to NDO due to spinal cord injury and multiple sclerosis [79], and they could modulate bladder afferent activity in patients with Parkinson’s disease and spinal cord injury [80]. BoNT-A injections into the bladder neck and urethral sphincter have been reported to alleviate voiding symptoms and increase the maximal flow rate in patients with lower urinary tract symptoms and a small prostate [81]. The intravesical administration of liposomal BoNT-A can be a simpler and less invasive delivery method for these patients, with a potential decrease in the risk of urinary retention or UTIs. In the future, advancements in both basic science and clinical research will be required to expand the clinical application of liposomal BoNT-A in functional bladder disorders.

## 6. Conclusions

Intravesical instillation using liposomes to encapsulate BoNT-A proteins is a potential treatment option to treat functional bladder disorders such as OAB, IC/BPS, and bladder oversensitivity. Although current clinical data regarding liposomal BoNT-A instillation are still limited in terms of its clinical efficacy on OAB and IC/BPS, adverse events due to intravesical BoNT-A injection can be avoided. With the future adjustment of the dose of BoNT-A, an increase in the instillation duration, and pretreatment with low-energy shock waves, liposome plus BoNT-A might play an important role in the treatment of bladder oversensitivity, OAB, and IC/BPS refractory to conventional medication.

## Data Availability

Not applicable.

## Abbreviations

The following abbreviations are used in this manuscript: AA: acetic acid; ACh: acetylcholine; ATP: adenosine triphosphate; AUA: American Urological Association; BoNT-A: botulinum toxin subtype A; CGRP: calcitonin-gene-related peptide; DMSO: dimethylsulfoxide; IC/BPS: interstitial cystitis/bladder pain syndrome; ICPI: Interstitial Cystitis Symptoms Index; ICSI: Interstitial Cystitis Problem Index; Liposomal BoNT-A: liposome-encapsulated BoNT-A; NDO: neurogenic detrusor overactivity; NGF: nerve growth factor; N/S: normal saline; OAB: overactive bladder; OABSS: Overactive Bladder Symptom Score; PVR: post-void residual; SNAP-25: synaptosomal-associated protein, 25 kDa; SUFU: Society for Urodynamics and Female Urology; SV2: synaptic vesicle protein 2; SV2A: synaptic vesicle protein 2A; TRPV1: transient receptor potential vanilloid receptor 1; UTI: urinary tract infection; UUI: urgency urinary incontinence.
